# Effect of aging on the transcriptomic changes associated with the expression of the HERV-K (HML-2) provirus at 1q22

**DOI:** 10.1186/s12979-020-00182-0

**Published:** 2020-05-13

**Authors:** Arttu Autio, Tapio Nevalainen, Binisha H. Mishra, Marja Jylhä, Heini Flinck, Mikko Hurme

**Affiliations:** 1 Faculty of Medicine and Health Technology, Tampere University, Arvo Ylpön katu 34, 33520 Tampere, Finland; 2 Gerontology Research Center (GEREC), Tampere, Finland; 3 Science Centre, Pirkanmaa Hospital District, Tampere, Finland; 4 Department of Clinical Chemistry, Faculty of Medicine and Health Technology, Tampere University, Tampere, Finland; 5 Finnish Cardiovascular Research Centre, Faculty of Medicine and Health Technology, Tampere University, Tampere, Finland; 6 Department of Clinical Chemistry, Fimlab Laboratories, Tampere, Finland; 7 Faculty of Social Sciences, Tampere University, Tampere, Finland; 8 Department of Clinical Microbiology, Fimlab Laboratories, Tampere, Finland

**Keywords:** Human endogenous retrovirus, HERV-K (HML-2), ERVK-7, Immunosenescence, GSEA, Next-generation sequencing, NGS, Gene ontology, Aging, Ageing

## Abstract

**Background:**

The human genome contains remnants of ancient retroviral infections called human endogenous retroviruses (HERV). Their expression is often observed in several diseases of autoimmune or inflammatory nature. However, the exact biological mechanisms induced by HERVs are still poorly understood. We have previously shown that several HERVs of the HERV-K (HML-2) family are strongly transcribed in the peripheral blood mononuclear cells (PBMC) derived from young and old individuals. To examine the potential functional consequences of HERV-K (HML-2) expression, we have now analyzed the correlation of its expression with age-associated changes in the transcriptome using gene set enrichment analysis (GSEA). We focused our analysis on the HERV-K (HML-2) provirus at 1q22, also known as ERVK-7.

**Results:**

The genes strongly correlating with the expression of HERV-K (HML-2) provirus at 1q22 expression were found to be almost entirely different in young and old individuals. The number of genes strongly correlating (Pearson correlation coefficient ≥ 0.7) with 1q22 expression was 946 genes in the old and 435 in the young, of which only 41 genes correlated strongly in both. Consequently, the related gene ontology (GO) biological processes were different. In the older individuals, many of the highest correlating processes relate to the function of neutrophils.

**Conclusions:**

The results of this work suggest that the biological processes associated with the expression of HERV-K (HML-2) provirus at 1q22 are different in the blood of young and old individuals. Specifically, a strong association was found in the older individuals between neutrophil activity and the expression of the HERV-K (HML-2) provirus at 1q22. These findings offer insight into potential effects of altered HERV expression in older individuals.

## Background

An estimated 8% of the human genome consists of endogenous retroviruses, which are remnants of exogenous retroviral infections that have integrated into the human germline throughout millions of years. The majority of these human endogenous retrovirus (HERV) proviruses do not contain intact open reading frames due to the accumulation of mutations, insertions, and deletions. Yet despite being seemingly harmless passengers, several studies have shown that activation of HERV proviruses is associated with various diseases, such as multiple sclerosis, rheumatoid arthritis and HIV infection (reviewed in [15]).

However, the exact immunomodulatory mechanisms of HERV activation are not known. It is clear that the original retrotranspositional mechanisms are not involved, as human ERVs have lost their infectious capacity. Therefore, it seems likely that the expressed HERV proteins (*env*, *gag*, *pol*) are critical in this respect [9]. HERV RNA could be recognized as a pathogen-associated molecular pattern (PAMP) by toll-like receptors, and this would induce type I interferon production contributing to the pathogenesis of autoinflammatory diseases [26].

**Fig. 1:**
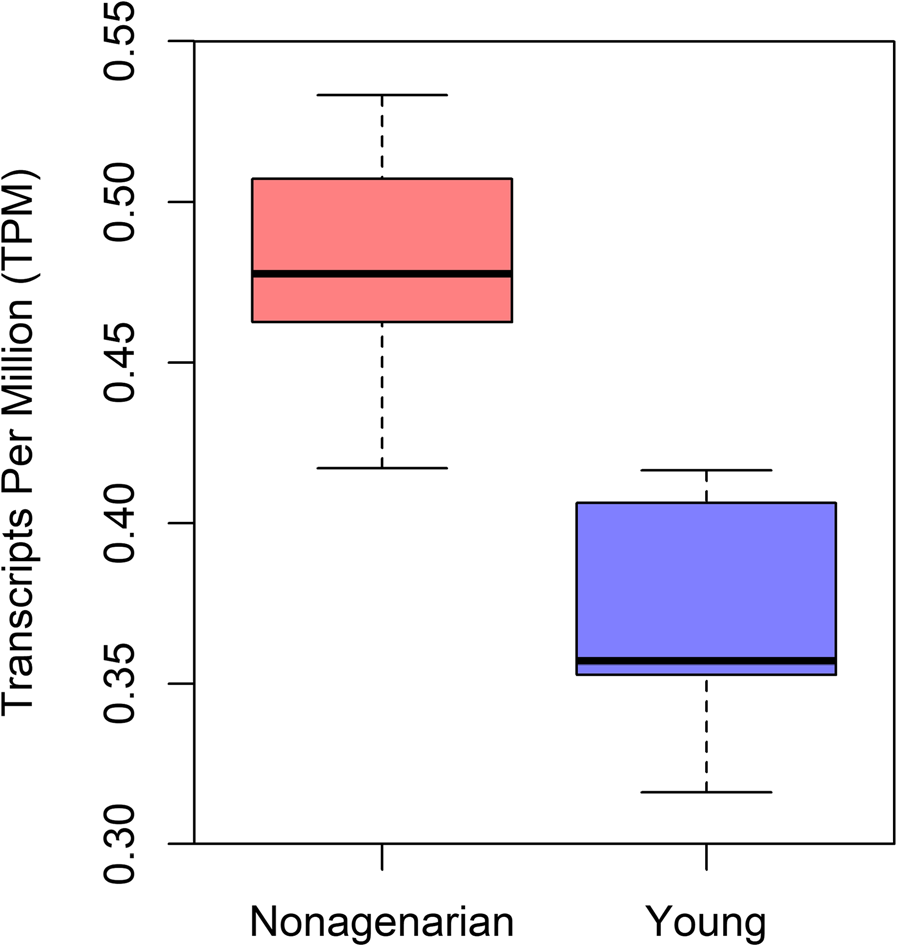
Relative expression of the HERV-K (HML-2) provirus at 1q22 in the nonagenarians compared to the young controls. Provirus at 1q22 had significantly differential expression (*p*-value < 0.05) between the age groups

The most recently integrated proviruses, belonging to the HERV-K (HML-2) family, are relatively well preserved and have retained considerable expression capacity [10]. Yet out of the 91 proviruses in the HERV-K family, only two, at 1q22 and at 1q23.3, are thought able to produce intact viral proteins [3]. In our previous work [16], we found the HERV-K (HML-2) at 1q22 to be significantly higher expressed (*p*-value < 0.05) in nonagenarian PBMCs (Peripheral Blood Mononuclear Cells) compared to young controls (Fig. 1), while expression of 1q23.3 was found to be very low. In our results, median 1q22 mRNA level in the young was 0.357 TPM (Transcripts Per Million), compared to 0.478 TPM in the old, and the mean 1q22 overexpression in the old was 1.3-fold. In this work, we have thus focused solely on the provirus at 1q22 in investigating the potential consequences of these transcriptomic changes. The provirus at 1q22 is also known as K102 and ERVK-7 [21].

## Results

In order to investigate the potential consequences of the significantly increased expression of HERV-K (HML-2) at 1q22 in PBMCs from older individuals, co-expression of the provirus with human genes was investigated utilizing Pearson’s product-moment correlation coefficient. Provirus 1q22 expression was found to correlate strongly (Pearson’s correlation coefficient ≥ 0.7) with the expression of 946 genes in nonagenarians, but with only 435 in the young controls, out of 9447 total genes. Of the genes that correlated strongly with 1q22 in either young or old individuals, only 41 correlated strongly (Pearson’s correlation coefficient ≥ 0.7) with 1q22 in both the young and the old. Highest correlating genes (Pearson’s correlation coefficient ≥ 0.95) can be seen in the supplementary file, in table S1. However, correlating the expression of one provirus to thousands of genes results in strong correlations with some genes by pure chance, even if there is no true association, and therefore it is not enough to look at individual correlations, but necessary to study these correlations as a whole.

To explore what biological processes might be affected by or be behind the transcriptomic changes, gene set enrichment analysis (GSEA) was performed based on known gene ontology (GO) functions of co-expressed genes [1, 20, 22]. Figure 2a and b show the 15 most upregulated and 15 most downregulated processes in both nonagenarians and young controls, respectively. The 20 most significantly associating GO biological processes are listed in Tables 1 and 2, in the order of significance, for both age groups. Figure 2c illustrates the strong association of 1q22 expression with genes involved in neutrophil activation in the older individuals, demonstrated by the number of highly correlating genes located at the beginning of the gene list.

**F ig. 2:**
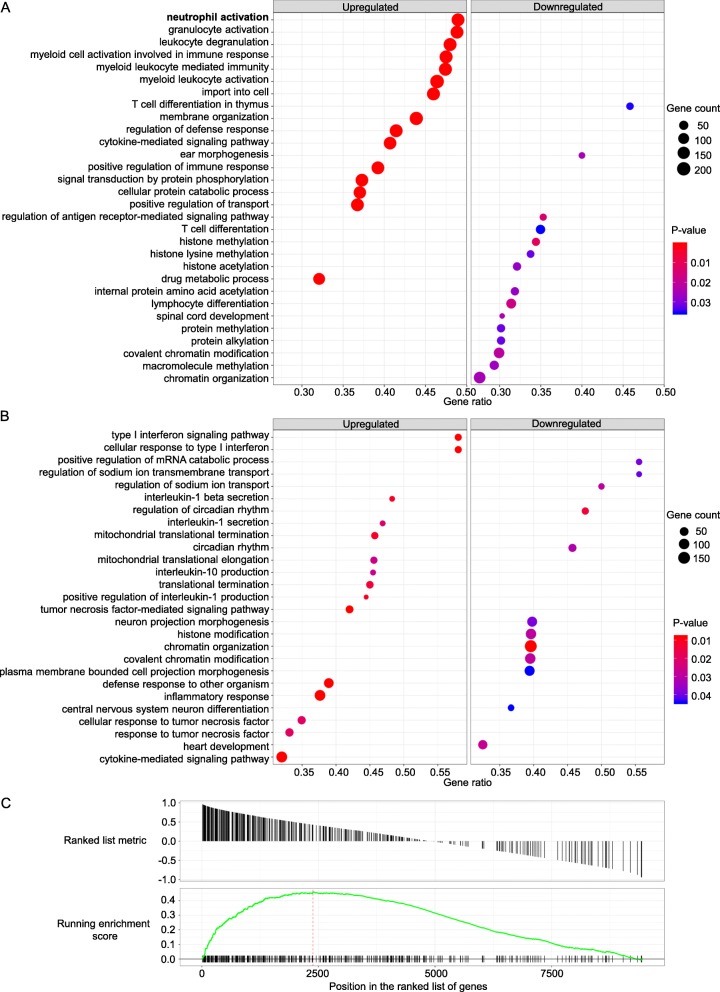
Results of the GSEA analysis. (**a** and **b**) Dot plots of GSEA results illustrating GO biological processes associated with HERV-K (HML-2) provirus 1q22 expression in both nonagenarian individuals and young controls respectively. The figures show the significant top 15 positively and the top 15 negatively enriched GO terms, based on co-expressed genes. There were only 12 significantly downregulated GO biological processes for the young. Gene count refers to the number of genes associated with each GO biological process. Gene ratio is the percentage of genes that significantly correlated with 1q22 expression from the total number of genes associated to that process. Terms are ranked in the figure by decreasing gene ratio. One GO term, “neutrophil activation”, directly involves neutrophil function and is bolded in the figure. (**c**) GSEA plot indicating enrichment of the biological process of neutrophil activation in the nonagenarians, based on the concentration of neutrophil activation –related genes at the beginning of the gene list ordered by correlation with 1q22 expression

**Table 1:** For nonagenarian samples, the GO biological processes that were most significantly associated with HERV-K (HML-2) provirus 1q22 expression, ordered by *p*-value. Enrichment score for each GO biological process is determined through GSEA, as shown in Fig. 2. Set size refers to the total number of the studied genes associated to each process, while gene ratio is the number of those genes that strongly correlated with 1q22 expression. The GO terms that are directly involved in neutrophil functions are in bold

ID	Description	*p*-value	Adjusted *p*-value	FDR (*q*-value)	Enrichment	Gene ratio	Set size
GO:0051050	positive regulation of transport	1.51E-05	7.07E-04	4.62E-04	0.283	37%	490
GO:0023014	signal transduction by protein phosphorylation	1.51E-05	7.07E-04	4.62E-04	0.262	37%	472
GO:0061024	membrane organization	1.51E-05	7.07E-04	4.62E-04	0.303	44%	467
GO:0050778	positive regulation of immune response	1.52E-05	7.07E-04	4.62E-04	0.297	39%	464
GO:0002274	myeloid leukocyte activation	1.52E-05	7.07E-04	4.62E-04	0.425	46%	459
GO:0044257	cellular protein catabolic process	1.52E-05	7.07E-04	4.62E-04	0.279	37%	451
GO:0031347	regulation of defense response	1.52E-05	7.07E-04	4.62E-04	0.287	41%	444
GO:0017144	drug metabolic process	1.53E-05	7.07E-04	4.62E-04	0.277	32%	430
GO:0019221	cytokine-mediated signaling pathway	1.53E-05	7.07E-04	4.62E-04	0.282	41%	425
GO:0002444	myeloid leukocyte mediated immunity	1.54E-05	7.07E-04	4.62E-04	0.448	47%	405
GO:0002275	myeloid cell activation involved in immune response	1.54E-05	7.07E-04	4.62E-04	0.447	47%	398
GO:0098657	import into cell	1.54E-05	7.07E-04	4.62E-04	0.334	46%	396
GO:0043299	leukocyte degranulation	1.54E-05	7.07E-04	4.62E-04	0.451	48%	394
GO:0036230	granulocyte activation	1.55E-05	7.07E-04	4.62E-04	0.462	49%	381
**GO:0042119**	**neutrophil activation**	**1.55E-05**	**7.07E-04**	**4.62E-04**	**0.461**	**49%**	**378**
**GO:0002446**	**neutrophil mediated immunity**	**1.55E-05**	**7.07E-04**	**4.62E-04**	**0.463**	**49%**	**377**
**GO:0002283**	**neutrophil activation involved in immune response**	**1.55E-05**	**7.07E-04**	**4.62E-04**	**0.466**	**49%**	**370**
**GO:0043312**	**neutrophil degranulation**	**1.55E-05**	**7.07E-04**	**4.62E-04**	**0.466**	**49%**	**370**
GO:0006897	endocytosis	1.55E-05	7.07E-04	4.62E-04	0.331	46%	367
GO:0002253	activation of immune response	1.56E-05	7.07E-04	4.62E-04	0.304	39%	368

**Table 2:** For young control samples, the GO biological processes that were most significantly associated with HERV-K (HML-2) provirus 1q22 expression, ordered by *p*-value. Enrichment score for each GO biological process is determined through GSEA, as shown in Fig. 2. Set size refers to the total number of the studied genes associated to each process, while gene ratio is the number of those genes that strongly correlated with 1q22 expression. No significantly enriched GO terms were directly involved in neutrophil function in the young control samples

ID	Description	*p*-value	Adjusted *p*-value	FDR (*q*-value)	Enrichment	Gene ratio	Set size
GO:0006325	chromatin organization	1.88E-05	8.19E-03	7.40E-03	−0.273	40%	475
GO:0060337	type I interferon signaling pathway	2.03E-05	8.19E-03	7.40E-03	0.491	58%	50
GO:0071357	cellular response to type I interferon	2.03E-05	8.19E-03	7.40E-03	0.491	58%	50
GO:0033209	tumor necrosis factor-mediated signaling pathway	2.06E-05	8.19E-03	7.40E-03	0.441	42%	100
GO:0098542	defense response to other organism	2.10E-05	8.19E-03	7.40E-03	0.324	39%	244
GO:0006954	inflammatory response	2.12E-05	8.19E-03	7.40E-03	0.280	38%	348
GO:0019221	cytokine-mediated signaling pathway	2.13E-05	8.19E-03	7.40E-03	0.299	32%	425
GO:0032732	positive regulation of interleukin-1 production	4.05E-05	1.10E-02	9.94E-03	0.584	44%	27
GO:0050702	interleukin-1 beta secretion	4.06E-05	1.10E-02	9.94E-03	0.561	48%	29
GO:0070126	mitochondrial translational termination	4.10E-05	1.10E-02	9.94E-03	0.439	46%	70
GO:0042752	regulation of circadian rhythm	5.87E-05	1.38E-02	1.24E-02	−0.453	49%	63
GO:0006415	translational termination	6.15E-05	1.38E-02	1.24E-02	0.412	45%	80
GO:0071356	cellular response to tumor necrosis factor	1.04E-04	1.99E-02	1.80E-02	0.347	35%	146
GO:0034612	response to tumor necrosis factor	1.04E-04	1.99E-02	1.80E-02	0.329	33%	160
GO:0050701	interleukin-1 secretion	1.22E-04	2.18E-02	1.97E-02	0.545	47%	32
GO:0032613	interleukin-10 production	1.62E-04	2.59E-02	2.34E-02	0.523	45%	33
GO:0070125	mitochondrial translational elongation	1.64E-04	2.59E-02	2.34E-02	0.429	46%	68
GO:0034340	response to type I interferon	1.83E-04	2.73E-02	2.46E-02	0.460	54%	54
GO:0032543	mitochondrial translation	2.06E-04	2.76E-02	2.49E-02	0.365	39%	101
GO:0007507	heart development	2.11E-04	2.76E-02	2.49E-02	−0.289	33%	240

To quantitatively assess the enrichment of neutrophil associated genes in the transcripts that are strongly co-expressed with 1q22, the hypergeometric test was utilized. The old individuals had 4 of the top 20 most significantly enriched GO terms involve neutrophils (Table 1), which according to the hypergeometric test results in a very low *p*-value of 4.96e-13, indicating significance (*p*-value < 0.05). This *p*-value signifies that the association to neutrophil function seen with 1q22 is very unlikely to be purely coincidental. For the young individuals, no significantly enriched biological processes were directly involved in neutrophil function (Table 2).

The GSEA indicated association between 1q22 and neutrophil activation was further studied by focusing on the correlations between 1q22 expression and common markers of neutrophil activation: IL-8 [29] and myeloperoxidase (MPO) [17]. The results are shown in Table 3. IL-8, also known as neutrophil chemotactic factor, had a strong and significant correlation with 1q22 in the old (Pearson’s correlation coefficient = 0.83, *p*-value = 0.02), but not in the young (Pearson’s correlation coefficient = 0.18, *p*-value = 0.70). In the old individuals, some correlation was seen between 1q22 and MPO, though this correlation was not statistically significant. MPO, along with human neutrophil elastase (HNE), is expressed in neutrophils and released to the extracellular space as part of neutrophil degranulation. The expression of HNE was below threshold (mean normalized read count < 50) and was therefore not included in analysis. Correlations between 1q22 and these genes were present only weakly in the PBMCs from young controls.

**Table 3:** Correlation of HERV-K (HML-2) at 1q22 to genes IL-8 and MPO that are common markers of neutrophil activity. Pearson’s correlation coefficient is used here. The correlation with IL-8 was statistically significant (*p*-value < 0.05) in the older individuals

Gene symbol	Correlation, old	*p*-value, old	Correlation, young	*p*-value, young
IL-8	0.832	0.020	0.180	0.699
MPO	0.509	0.244	0.247	0.593

## Discussion

Many of the biological processes enriched in the PBMCs from the old individuals are related to neutrophil activation and function (Fig. 2a and Table 1). This association between 1q22 expression and neutrophil function is not present in the samples from young controls. In addition to the differences between age groups in the number of directly neutrophil involving GO terms, the GSEA results for the old contain many GO terms that are indirectly related to neutrophil function, such as terms describing granulocyte and leukocyte activity as well as more general immune system functions. Elevated levels of neutrophil activation in old individuals have already been previously reported. For example, in a time series study spanning 15 years, Samson et al. found that the numbers of neutrophils and monocytes increased with age, correlating with frailty and CRP trajectories [18].

It is known that neutrophils are crucial in the first line of defense against infectious agents and that several of their functional activities are affected by aging [7]. As host cells for viruses, neutrophils are suboptimal, as they are both short-lived and terminally differentiated. However, internalization of viruses during infection does occur, for example with the West Nile virus, which is reported to replicate within neutrophils [2].

Though many of the weapons in the neutrophil’s arsenal are specifically for bacteria, neutrophils still play an important indirect role in viral infection defense by influencing the behavior of other cell subsets [19]. It is this indirect relationship between neutrophils and viruses that may explain why the results indicate enrichment of neutrophil-associated functions in PBMC samples that should not contain more than trace amounts of neutrophil-derived RNA. Though neutrophils are absent from the samples, the effects exerted by lymphocytes on neutrophils and by neutrophils on lymphocytes can still be seen in gene expression from the lymphocyte-containing PBMC samples. For example, neutrophil chemotaxis and activation can be induced by lymphocyte-produced IL-8 [29]. In the results of this work, IL-8 expression was found to strongly correlate with 1q22 expression in the old, yet not in the young, which could be seen as another link between 1q22 expression and neutrophil activation.

Based on the results of this work, it could thus be speculated that a chronic activation of neutrophils by HERVs is leading to the functional exhaustion of neutrophils as part of the aging-associated decline of immune capacity, i.e., immunosenescence. Additionally, as the expression of various HERVs have been shown to be increased in several diseases of autoimmune and autoinflammatory nature, HERV expression could be leading to increased production of pro-inflammatory mediators and in this way potentiating age-associated inflammation, referred to as “inflammaging”. It would therefore be of interest to analyze HERV-associated biological processes in cases of autoimmune or autoinflammatory disease.

If HERVs were indeed causing activation of neutrophil-associated mechanisms, they would not be the only viruses to do so. Dunning et al. [6] described a comparable change in the analysis of the blood transcriptome in patients with long-lasting and severe influenza infection. This change from the “anti-viral” transcriptomic signature (including the effects of type-I interferons) to the “anti-bacterial” signature (e.g. neutrophil-mediated) is similar to that we observed in the HERV-K 1q22 expressing PBMC from the older individuals. However, Dunning et al. [6] saw a rise in transcripts associated with the GO term “response to bacterium”, while that GO term was not enriched in our data. Additionally the transcripts that we found to most strongly correlate with 1q22 in young and in old separately (Supplementary file, Table S1) had no overlap with the transcripts that Dunning et al. describe to be associated with the GO terms “response to virus” or “response to bacterium”. The difference in results could be explained by Dunning et al. having sequenced whole-blood samples, as opposed to the PBMC samples used in this work. In our results, the association with neutrophils does not come from a general response to bacterium, rather we see a more specific co-expression with transcripts associated with neutrophil activation.

As a limitation of this work, the cell types involved in this provirus 1q22 associated processes cannot be determined based on this PBMC derived data, as the correlating transcripts could be derived from different cell types. A natural next step in this line of research would therefore be to investigate this association between 1q22 and neutrophils in datasets from specific cell types.

## Conclusions

The results of this work suggest that the biological processes associated with the expression of HERV-K (HML-2) provirus at 1q22 are different in the blood of young and old individuals. The number of genes strongly correlating with the expression of the provirus is higher in the PBMC from the old individuals compared to the young, and the associated GO biological processes differ between the age groups. The strong association that was found in the older individuals between neutrophil activity and the expression of HERV-K (HML-2) provirus at 1q22 could be explained by a chronic activation of neutrophils by HERVs, leading to the functional exhaustion of neutrophils as part of immunosenescence. Expression f this provirus could also be leading to increased production of pro-inflammatory mediators, thus potentiating inflammaging. Taken as a whole, these findings offer insight into potential effects of altered HERV expression in older individuals. Investigation of provirus 1q22 expression associated biological functions in specific cell types and in conditions of autoimmune or autoinflammatory disease could further elucidate the consequences of its expression.

## Materials and methods

This work extends the bioinformatic analyses done on our Vitality 90+ dataset that we have previously presented in [16]. Below are again described the sample collection and RNA sequencing protocols used, followed by the new analyses that have been performed as part of this work.

### Study populations

Samples were collected from two populations: nonagenarian individuals (*n* = 7) and young controls (*n* = 7). Nonagenarians studied are all 94-year-old women, participants in The Vitality 90+ study. Young controls are women aged between 26 and 32, with a median age of 28, all healthy laboratory personnel with no medically diagnosed chronic illnesses, were non-smokers and had not had any infections or received any vaccinations within the 2 weeks prior to blood sample collection. The methods of recruitment and characterization of participants were done as has been reported previously [8]. The study participants provided their written informed consent.

### Sample collection

Blood samples were collected by a trained laboratory technician in laboratory facilities. All blood samples were drawn between 8 am and 12 am and collected into EDTA containing tubes. Samples were directly subjected to leucocyte separation on a Ficoll-Paque density gradient (Ficoll-Paque™ Premium, cat. no. 17-5442-03, GE Healthcare Bio-Sciences AB, Uppsala, Sweden). The PBMC layer was collected and cells used for RNA extraction were suspended in 150 μl of RNAlater solution (Ambion Inc., Austin, TX, USA).

### RNA extraction

RNA used for RNA sequencing was purified using a miRNeasy mini kit (Qiagen, CA, USA) according to manufacturer’s protocol with on-column DNA digestion (Qiagen). The concentration and quality of the RNA was assessed with a NanoDrop ND-1000 spectrophotometer (NanoDrop Technologies, Wilmington, DE, USA).

### RNA sequencing

Agilent Bioanalyzer RNA nano chips (Agilent) were used to evaluate the integrity of total RNA and Qubit RNA kit (Life Technologies) to quantitate RNA in samples. 1 μg of total RNA was used for ScriptSeq™ Complete Gold System (Epicentre) to ribodeplete rRNA and further for RNA-seq library preparation. SPRI beads (Agencourt AMPure XP, Beckman Coulter) were used for purification of RNAseq libraries. The library QC was evaluated on High Sensitivity chips by Agilent Bioanalyzer (Agilent). Paired-end sequencing of RNA-seq libraries was done using Illumina HiSeq technology with a minimum of 60 million 2x100bp paired-end reads per sample.

### Data preprocessing

Raw reads were aligned to human genome reference build hg19 using TopHat v2.0.13 [24] with the default parameters. SAMtools [12] was used to filter out reads mapping to multiple regions of the genome. The raw expression estimates were calculated using the Cuffquant tool from Cufflinks v.2.2.1 [23, 25]. The expression values were normalized with the Cuffnorm tool from Cufflinks v.2.2.1 [23, 25], utilizing the geometric normalization method, which scales the read counts as well as the FPKM values according to procedure described in Love et al. [14]. To ensure the robustness of the normalization the expressions of HERV elements were quantified and normalized together with ENSEMBL v. 82 gene reference set [11, 27]. The annotation data for HERV-K (HML-2) was from Subramanian et al. [21].

### Correlation calculations

To prepare the dataset for correlation analysis, low expression genes with a mean normalized read count lower than 50 were filtered out. This threshold reduced the number of transcripts in the dataset from 57,886 to 12,639. Genes with no associated GO terms were also removed, utilizing R package msigdbr [13] (version 7.0.1), further reducing the number of genes to 9447. Changes to the mean transcript counts as a result of the filtering are shown in Table 4. Correlations between the expressions of each gene and of HERV-K (HML-2) at 1q22 were calculated utilizing Pearson’s product-moment correlation coefficient. The R programming language (version 3.6.1) with stats package was used to calculate the correlation coefficients.

**Table 4:** Mean normalized transcript read count quantiles before and after filtering out low expression genes and genes with no associated GO terms

	0%	25%	50%	75%	100%
Before filtering	0.00	0.00	0.00	23.61	598,428.00
After filtering	50.00	211.22	595.69	1512.92	239,124.66

The expression of the filtered genes across samples approximates normal distribution in accordance with the assumptions of the Pearson correlation coefficient significance calculation. The supplementary file figures S1 and S2 show Q-Q plots of 1q22 expression against normal distribution in the nonagenarian and young control samples respectively.

### GSEA

Gene set enrichment analysis (GSEA) was used to evaluate the potential biological functions of the HERV-K (HML-2) provirus at 1q22. The gene list used in GSEA was ranked by Pearson correlation coefficient of the expression between 1q22 and genes. The GSEA function used here is provided by the clusterProfiler software package version 3.12.0 [28], which utilizes biological process (BP) gene ontology (GO) terms [1, 22]. The genome annotation information was retrieved with R package org.Hs.eg.db [4] (version 3.8.2). In the GSEA, the minimum size of gene sets was 25 genes and the maximum was 500. Permutation size was 100,000. Significance of enrichment was determined by a Benjamini-Hochberg corrected *p*-value of less than 0.05.

### Hypergeometric test

In order to statistically study the incidence of neutrophil related GO terms in the top 20 most significantly enriched terms, the hypergeometric test was utilized. The R programming language [5] (version 3.6.1) and more specifically the packages stats and GO.db were used to perform the hypergeometric test. There was a total of 29,698 biological process GO terms, of which only 17 specifically describe neutrophil functions. For the old individuals, 4 GO terms related to neutrophil function were found within the top 20 most significantly enriched GO terms. For the young individuals, no significantly enriched GO terms were related to neutrophils.

## Supplementary information

**Additional file 1: Table S1.** Genes that had the highest correlation (Pearson correlation coefficient > 0.95) with 1q22 expression. Both positive and negative correlation are included. There is no overlap in these genes between the age groups. **Figure S1.** Q-Q plot of the HERV-K (HML-2) provirus at 1q22 expression across nonagenarian samples against normal distribution. The plot as well as a Shapiro-Wilk test (*p*-value = 0.58) support that the distribution of the expression of 1q22 in the studied samples approximates normal distribution. **Figure S2.** Q-Q plot of the HERV-K (HML-2) provirus at 1q22 expression across young samples against normal distribution. The plot as well as a Shapiro-Wilk test (*p*-value = 0.32) support that the distribution of the expression of 1q22 in the studied samples approximates normal distribution.

## Data Availability

The datasets previously generated [13] and further analyzed in this work are available in the Gene Expression Omnibus (GEO) repository (https://www.ncbi.nlm.nih.gov/geo/), with accession number GSE122309.

## Abbreviations

ERV: endogenous retrovirus
GO: gene ontology
GSEA: gene set enrichment analysis
HERV: human endogenous retrovirus
PAMP: pathogen-associated molecular pattern
PBMC: peripheral blood mononuclear cell
TPM: transcripts per million
