# Supplementary material for: Effect of aging on the transcriptomic changes associated with the expression of the HERV-K (HML-2) provirus at 1q22
Source: Immun Ageing. 2020 May 13;17:11. doi: 10.1186/s12979-020-00182-0 (PMC7218820; doi:10.1186/s12979-020-00182-0)

**Supplementary file**

**Table S1.** Genes that had the highest correlation (Pearson correlation coefficient >0.95) with 1q22 expression. Both positive and negative correlation are included. There is no overlap in these genes between the age groups.

Panel A: Nonagenarians Panel B: Young controls

|  | **Ensembl gene ID** | **Gene symbol** | **Correlation** |  |  | **Ensembl gene ID** | **Gene symbol** | **Correlation** |
| --- | --- | --- | --- | --- | --- | --- | --- | --- |
| 1 | ENSG00000104447 | TRPS1 | 0.995 |  | 1 | ENSG00000137078 | SIT1 | 0.979 |
| 2 | ENSG00000198915 | RASGEF1A | -0.994 |  | 2 | ENSG00000180071 | ANKRD18A | 0.978 |
| 3 | ENSG00000113494 | PRLR | 0.984 |  | 3 | ENSG00000177570 | SAMD12 | 0.969 |
| 4 | ENSG00000132205 | EMILIN2 | 0.984 |  | 4 | ENSG00000148481 | MINDY3 | -0.968 |
| 5 | ENSG00000059377 | TBXAS1 | 0.982 |  | 5 | ENSG00000112996 | MRPS30 | 0.967 |
| 6 | ENSG00000140474 |  | -0.980 |  | 6 | ENSG00000172673 | THEMIS | -0.966 |
| 7 | ENSG00000144959 | NCEH1 | 0.979 |  | 7 | ENSG00000197162 | ZNF785 | 0.965 |
| 8 | ENSG00000134287 | ARF3 | 0.979 |  | 8 | ENSG00000110367 | DDX6 | -0.956 |
| 9 | ENSG00000126067 | PSMB2 | 0.970 |  | 9 | ENSG00000119185 | ITGB1BP1 | -0.951 |
| 10 | ENSG00000112531 | QKI | 0.968 |  | 10 | ENSG00000170734 | POLH | 0.951 |
| 11 | ENSG00000015676 | NUDCD3 | -0.967 |  |  |  |  |  |
| 12 | ENSG00000118564 | FBXL5 | 0.967 |  |  |  |  |  |
| 13 | ENSG00000125703 | ATG4C | 0.966 |  |  |  |  |  |
| 14 | ENSG00000108100 | CCNY | 0.965 |  |  |  |  |  |
| 15 | ENSG00000134755 | DSC2 | 0.965 |  |  |  |  |  |
| 16 | ENSG00000038427 | VCAN | 0.962 |  |  |  |  |  |
| 17 | ENSG00000140740 | UQCRC2 | 0.962 |  |  |  |  |  |
| 18 | ENSG00000108239 | TBC1D12 | 0.961 |  |  |  |  |  |
| 19 | ENSG00000196352 | CD55 | 0.960 |  |  |  |  |  |
| 20 | ENSG00000184441 |  | -0.959 |  |  |  |  |  |
| 21 | ENSG00000084234 | APLP2 | 0.959 |  |  |  |  |  |
| 22 | ENSG00000115762 | PLEKHB2 | 0.958 |  |  |  |  |  |
| 23 | ENSG00000109323 | MANBA | 0.957 |  |  |  |  |  |
| 24 | ENSG00000107185 | RGP1 | -0.957 |  |  |  |  |  |
| 25 | ENSG00000013523 | ANGEL1 | 0.957 |  |  |  |  |  |
| 26 | ENSG00000168615 | ADAM9 | 0.956 |  |  |  |  |  |
| 27 | ENSG00000188921 | HACD4 | 0.956 |  |  |  |  |  |
| 28 | ENSG00000108953 | YWHAE | 0.956 |  |  |  |  |  |
| 29 | ENSG00000005893 | LAMP2 | 0.956 |  |  |  |  |  |
| 30 | ENSG00000078369 | GNB1 | 0.955 |  |  |  |  |  |
| 31 | ENSG00000067208 | EVI5 | 0.955 |  |  |  |  |  |
| 32 | ENSG00000074935 | TUBE1 | -0.954 |  |  |  |  |  |
| 33 | ENSG00000132932 | ATP8A2 | -0.954 |  |  |  |  |  |
| 34 | ENSG00000068831 | RASGRP2 | -0.954 |  |  |  |  |  |
| 35 | ENSG00000121413 | ZSCAN18 | -0.952 |  |  |  |  |  |
| 36 | ENSG00000035681 | NSMAF | 0.952 |  |  |  |  |  |
| 37 | ENSG00000147168 | IL2RG | -0.952 |  |  |  |  |  |
| 38 | ENSG00000188171 | ZNF626 | -0.952 |  |  |  |  |  |
| 39 | ENSG00000115840 | SLC25A12 | -0.951 |  |  |  |  |  |
| 40 | ENSG00000109911 | ELP4 | 0.951 |  |  |  |  |  |
| 41 | ENSG00000162711 | NLRP3 | 0.951 |  |  |  |  |  |
| 42 | ENSG00000138829 | FBN2 | 0.951 |  |  |  |  |  |
| 43 | ENSG00000008083 | JARID2 | 0.951 |  |  |  |  |  |
| 44 | ENSG00000023318 | ERP44 | 0.950 |  |  |  |  |  |
| 45 | ENSG00000119900 | OGFRL1 | 0.950 |  |  |  |  |  |
| 46 | ENSG00000114450 | GNB4 | 0.950 |  |  |  |  |  |
| 47 | ENSG00000079277 | MKNK1 | 0.950 |  |  |  |  |  |

**Figure S1.** Q-Q plot of the HERV-K (HML-2) provirus at 1q22 expression across nonagenarian samples against normal distribution. The plot as well as a Shapiro-Wilk test (p-value = 0.58) support that the distribution of the expression of 1q22 in the studied samples approximates normal distribution.


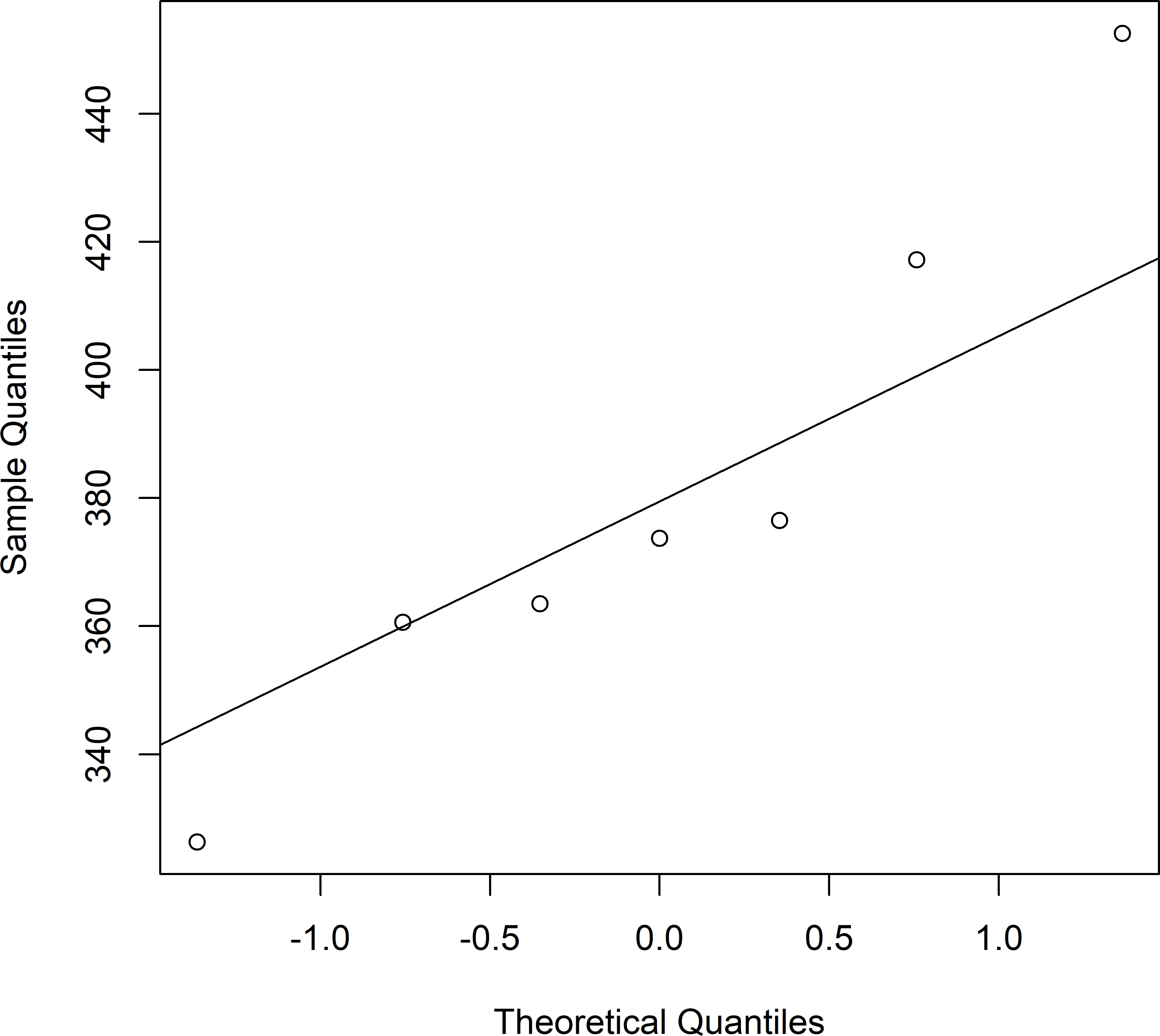


**Figure S2.** Q-Q plot of the HERV-K (HML-2) provirus at 1q22 expression across young samples against normal distribution. The plot as well as a Shapiro-Wilk test (p-value = 0.32) support that the distribution of the expression of 1q22 in the studied samples approximates normal distribution.


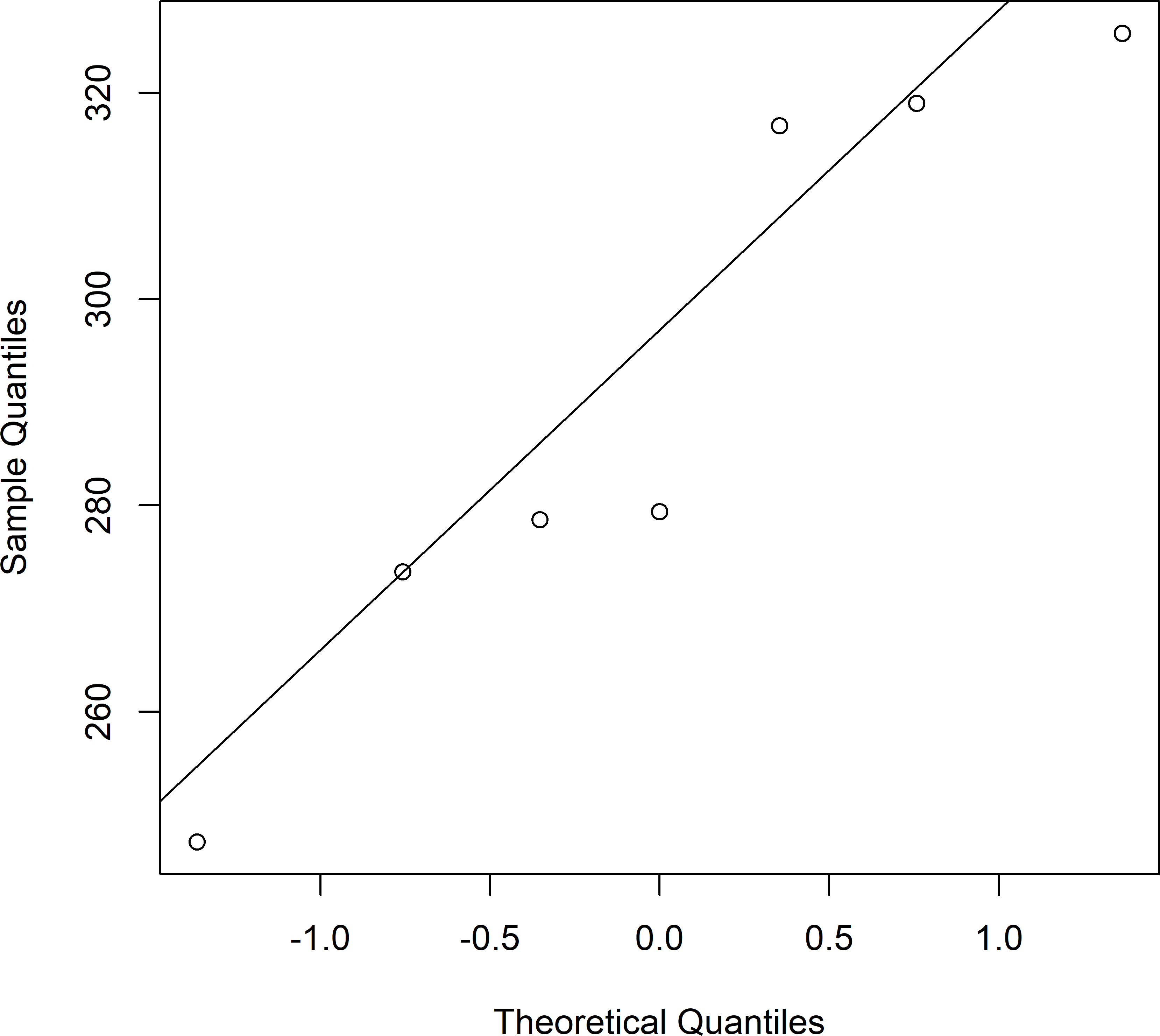

Supplement: Supplementary file 1 — Additional file 1: Table S1. Genes that had the highest correlation (Pearson correlation coefficient > 0.95) with 1q22 expression. Both positive and negative correlation are included. There is no overlap in these genes between the age groups. Figure S1. Q-Q plot of the HERV-K (HML-2) provirus at 1q22 expression across nonagenarian samples against normal distribution. The plot as well as a Shapiro-Wilk test (p-value = 0.58) support that the distribution of the expression of 1q22 in the studied samples approximates normal distribution. Figure S2. Q-Q plot of the HERV-K (HML-2) provirus at 1q22 expression across young samples against normal distribution. The plot as well as a Shapiro-Wilk test (p-value = 0.32) support that the distribution of the expression of 1q22 in the studied samples approximates normal distribution. [file 12979_2020_182_MOESM1_ESM.docx]
